# COVID-19 vaccination survey and anti-SARS-CoV-2 IgG responses in a human cohort from *Schistosoma mansoni*-endemic villages in Mayuge District, Uganda: a cross-sectional study

**DOI:** 10.3389/fpubh.2024.1437063

**Published:** 2024-11-18

**Authors:** Mimi Niu, Yi Mu, Moses Adriko, Rowel Candia, Malcolm K. Jones, Donald P. McManus, Thomas G. Egwang, Pengfei Cai

**Affiliations:** ^1^Molecular Parasitology Laboratory, QIMR Berghofer Medical Research Institute, Brisbane, QLD, Australia; ^2^School of Life Science, Hainan University, Haikou, China; ^3^Vector Borne and Neglected Tropical Disease Control Division, Ministry of Health, Kampala, Uganda; ^4^School of Veterinary Science, The University of Queensland, Brisbane, QLD, Australia; ^5^Department of Immunology and Parasitology, Med Biotech Laboratories, Kampala, Uganda; ^6^School of Biomedical Sciences, The University of Queensland, Brisbane, QLD, Australia

**Keywords:** COVID-19, SARS-CoV-2, coronavirus, schistosomiasis, *Schistosoma mansoni*, helminth, vaccine, Uganda

## Abstract

**Introduction:**

The coronavirus disease 2019 (COVID-19) pandemic has resulted in devastating health and economic consequences worldwide. Vaccination has been a central pillar for COVID-19 prevention and control. Understanding the immunomodulatory effects of helminth infections on COVID-19 vaccine-induced immune responses and vaccine efficacy is crucial to the development and deployment of effective vaccination strategies in low- and middle-income countries with a high prevalence of worms.

**Methods:**

In September 2022, we conducted a cross-sectional, population-based survey in five *Schistosoma mansoni* endemic villages in Mayuge District, Uganda (*n* = 450). The prevalence of schistosomiasis and soil-transmitted helminths was determined by the Kato-Katz (KK) technique on two stool samples collected from each participant. A subset of individuals (*n* = 204) were interviewed in a COVID-19 vaccination survey. IgG levels against the SARS-CoV-2 spike S1 subunit (anti-S1 IgG) were measured by enzyme-linked immunosorbent assay (ELISA) in collected serum samples.

**Results:**

The overall schistosomiasis and hookworm prevalence rates in the five villages were 36.4% (166/450) and 36.9% (168/450), respectively. Within the cohort, 69.78% (314/450) of the subjects had a positive anti-S1 IgG response. COVID-19 vaccination coverage among the interviewed participants was 93.14% (190/204; 95% CI, 88.8% − 95.9%). However, 81% (154/190) of COVID-19 vaccinees had an anti-S1 IgG titre ≤200. In an adolescent group receiving a single dose of the BNT162b2 mRNA vaccine (*n* = 23), an inverse correlation was observed between anti-S1 IgG antibody level/titre and faecal egg count. Within the above group, anti-S1 IgG levels/titres were significantly lower in subjects with moderate or heavy *S. mansoni* infection (*n* = 5) than those in KK-negative individuals (*n* = 9).

**Conclusion:**

Although the acceptance rate of COVID-19 vaccination was high, the majority of participants received only a single vaccine dose and the overall anti-S1 IgG titres in confirmed vaccinees were low. Moderate-to-heavy schistosome infections blunted the antibody responses following vaccination with a single dose of BNT162b2. These observations confirm the necessity for a second COVID-19 vaccine dose for two-dose primary immunization series and call for implementation research that may inform the development of a ‘treat and vaccinate’ policy during vaccination roll-out in regions with heavy worm burdens.

## 1 Introduction

Coronavirus disease 2019 (COVID-19), caused by severe acute respiratory syndrome coronavirus 2 (SARS-CoV-2), affects more than 700 million people and has caused more than 7.3 million reported deaths worldwide (1). Uganda experienced three major waves of COVID-19 in mid-late 2020, April – August 2021, and December 2021 – February 2022, respectively (2). As of 23 October 2022, a total of 169,473 confirmed cases and 3,630 deaths had been reported (3), resulting in an infection rate of approximately 0.3%. Some studies reported the presence of pre-existing SARS-CoV-2 cross-reactive immunological responses in Uganda populations (4, 5). While a previous infection with a common human coronavirus (hCoV) may enhance the immune response to SARS-CoV-2 infection, such ‘cross-reactive immunity’ decreases with age (6, 7). Previous hCoV exposure may also cause serological cross-reactivity with SARS-CoV-2 antigens, with cross-reactive IgG was more common against the nucleoprotein than the spike (5, 8, 9). A COVID-19 vaccination campaign was launched in Uganda on 10 March 2021 (10). A number of COVID-19 vaccines including ChAdOx1 nCoV-19 (AstraZeneca), Ad26.COV2.S (Janssen), CoronaVac (Sinovac), BNT162b2 (Pfizer-BioNTech) and mRNA-1273 (Moderna) have been administered in the country. Most of these, such as ChAdOx1 nCoV-19, Ad26.COV2.S, and BNT162b2 elicit Th1-biased cellular immune responses (11–13). By 26 November 2023, 44% of the total population in Uganda was vaccinated with at least one dose of a COVID-19 vaccine, whereas 29% of the population was vaccinated with a complete primary series of a COVID-19 vaccine (1).

Helminths are extremely common and highly prevalent infections of humans in low- and middle-income countries (LMICs), where coinfection with helminths and SARS-CoV-2 may occur. Coinfections may increase in these countries because the basic medical services and government resources needed for anti-helminth public health campaigns have been diverted for COVID-19 during the pandemic. Chronic infections with soil-transmitted helminths (STHs), schistosomes and filarial worms in LMICs alter the immune system to a type 2 (Th2)-skewed immunity (14) together with Treg-mediated immune responses (15). Some evidence suggests that prior helminth infection may impair host immune responses to vaccines against other types of pathogens (16–21). However, other studies have shown that helminth infection has a limited influence on the immunogenicity of vaccines (22, 23). There has been a call for the scientific research community and funding agencies to support clinical trials of COVID-19 vaccines in people with helminth infections as a priority population that represents a quarter of humanity (24).

Uganda has a high prevalence of schistosomiasis and other helminthiasis (25), which may affect immune responses in endemic communities following COVID-19 vaccination. However, no previous studies of COVID-19 vaccination coverage and vaccine-induced antibody responses have been reported in rural schistosomiasis-endemic areas of Uganda. In this study, we recruited 450 subjects from five schistosomiasis-endemic villages in Mayuge District to conduct a survey on the prevalence of schistosomiasis and soil-transmitted helminthiasis and COVID-19 vaccine coverage. As the SARS-CoV-2 spike protein, particularly the S1 subunit (S1), is the primary target for eliciting neutralizing antibody responses (26), we measured IgG antibodies against the S1 subunit using serum samples collected from participants. Overall, the three primary objectives of the study were (i) to assess the COVID-19 vaccination status in rural schistosomiasis endemic areas; (ii) to estimate the approximate distribution of antibody levels against the S1 protein in COVID-19 vaccinees; and (iii) to investigate the effects of schistosome infection intensity on serological responses against the S1 subunit in COVID-19 vaccinees.

## 2 Methods

### 2.1 Ethics

Ethical approval for the study was obtained from the Institutional Review Board, Vector Control Division, Ministry of Health, Uganda (IRB number VCDREC160) and the Human Ethics Committee of QIMR Berghofer Medical Research Institute (QIMRB) (project number: P3700). All participants provided written informed consent (for individuals aged 18 years or under, consent was obtained from their legal guardians prior to the administration of the survey and sampling). The research protocol was approved by the Uganda National Council for Science and Technology (UNCST) (ref: UNCST/INV/870).

### 2.2 Study design and period

A representative descriptive and analytical cross-sectional study was conducted from 16 to 28 September 2022. By October 2022, two COVID-19 epidemic waves had occurred in Uganda, with a total of 169,473 confirmed cases and 3,630 deaths (3).

### 2.3 Setting

Uganda is an East African country with a total area of 241,038 km^2^ and an estimated population of 49.6 million in 2023. The country’s gross domestic product *per capita* was 3,640 international dollars in 2024 (27). Its human development index stands at 0.55 as of 2022, positioning it at 159 out of 193 countries and territories (28).

### 2.4 Study population

**Figure 1:**
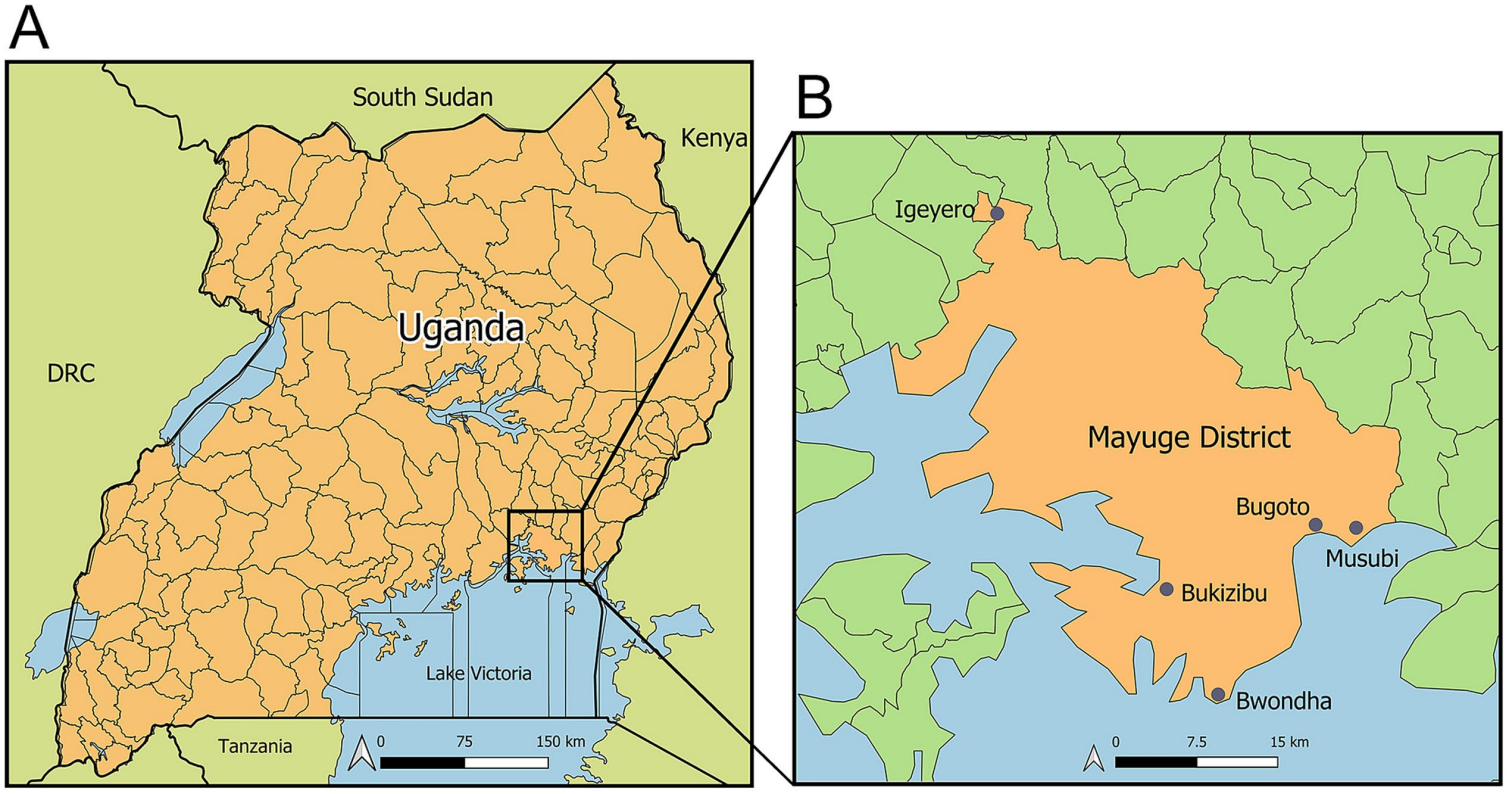
Maps showing the geographical locations of the study sites. (A) Map of Uganda; (B) Map of Mayuge District showing the five study villages: Bugoto, Bwondha, Musubi, Bukizibu, and Igeyero. Created using QGIS 3.32 software (QGIS Association. QGIS Geographic Information System. 3.32 ed2023).

The target population was the residents of five villages in Mayuge District, including Bugoto, Bwondha, Musubi, Bukizibu, and Igeyero (Figure 1). The inclusion criteria included: (a) being over 5 years of age; (b) providing informed consent or assent to participate in the study and to provide two stool samples and one blood sample. A total of 450 participants were enrolled.

### 2.5 Data and sample collection

Data collection was carried out by qualified research staff trained in survey procedures. Data were collected from consenting participants during interviews via a structured questionnaire to elicit information on socio-demographic characteristics, deworming history, attitudes and practices related to schistosomiasis. Information on COVID-19 vaccination status was also collected from a subset of participants (*n* = 204).

Two stool samples were collected from each individual on two separate days, for Kato-Katz (KK) detection (3 slides per stool sample) of eggs of *S. mansoni*, hookworm, *Trichuris trichiura*, *Ascaris lumbricoides* and other helminths.

A 10 mL peripheral venous blood sample was collected from each participant in blue-top sodium citrate tubes and allowed to clot for 30 min at room temperature. Whole blood samples were stored in a container at 4°C and then transferred to the laboratory, where serum samples were obtained after centrifugation at 1,500 × *g* for 10 min. The samples were then aliquoted (~1.5 mL per aliquot) into three cryovials and stored at −20°C before being shipped on dry ice to the QIMRB for further analysis. A material transfer agreement (MTA) was established prior to sample transfer.

### 2.6 Serological analyses

Serum samples were kept frozen at −80°C until analysis. An enzyme-linked immunosorbent assay (ELISA) was used to determine the levels/titres of serum IgG antibody to the SARS-CoV-2 spike S1 protein (anti-S1 IgG), as previously described with minor modifications (29, 30). Briefly, recombinant SARS-CoV-2 S1 protein (aa14-683, accession number: YP_009724390.1) (Cat# RP87681; Thermo Fisher Scientific, Australia) was coated on 96-well MaxiSorp plates at a concentration of 1 ng/μL (100 μL/well) in coating buffer overnight at 4°C. The plates were washed once with PBST solution (phosphate-buffered saline, pH 7.5, containing 0.05% Tween-20) and incubated with blocking buffer (PBST containing 1% BSA) at 37°C for 1 h. For anti-S1 IgG detection in the cohort, serum samples were diluted 1:100 in blocking buffer. For anti-S1 IgG titre determination, serial two-fold dilutions of sera were prepared from 1:100 to 1:32,00. After 100 μL of diluted serum sample was added to each well, the plates were incubated at 37°C for 1 h followed by the addition of a mouse monoclonal anti-human IgG (Fc specific)-biotin antibody (Cat# B3773, Merck Life Science, Australia) as a secondary antibody (1:160,000, 100 μL/well) at 37°C for 1 h. The plates were then incubated with streptavidin-HRP (Cat# 554066, BD Biosciences, CA, USA) (1:10,000, 100 μL/well) to detect antibodies bound to the S1 protein. The plates were washed 5 times with wash buffer after each step following the addition of serum. TMB substrate (100 μL) (Cat# 860336; Merck Life Science, Australia) was then added to the plates and the reactions were terminated by adding 50 μL of stop solution (2 N H_2_SO_4_). The optical density (OD) was measured at 450 nm using a microplate spectrophotometer (PowerWave XS2, BioTek, VT, USA). Pre-pandemic sera collected from Heilongjiang Province, China, before the outbreak of COVID-19 served as negative controls (*n* = 20) (31, 32). The cut-off value for a positive reaction was calculated as 2.1 multiplied by the mean of the OD_450_ values of negative controls.

To assess the serum distribution of anti-S1 IgG subclasses, MaxiSorp plates were coated with the S1 recombinant protein (100 ng/well). The plates were incubated overnight at 4°C and blocked with blocking buffer for 1 h at 37°C. Serum samples were tested at a dilution of 1:100. The following mouse anti-human IgG secondary antibodies were used: IgG1, (1:500, Cat# A-10648; Thermo Fisher Scientific, Australia), IgG2, (1:500, Cat# 05–0520; Thermo Fisher Scientific, Australia), IgG3 (1:500: Cat# 05–3,620; Thermo Fisher Scientific, Australia), and IgG4 1:500 (Cat# A-10654; Thermo Fisher Scientific, Australia).

### 2.7 Statistical analysis

Statistical data were analysed using GraphPad Prism (v.9) for Windows (GraphPad Software, Inc., San Diego, CA, USA). IgG titres were natural log transformed. The Mann–Whitney *U* test, the Kruskal-Wallis test followed by Dunn’s multiple comparison or one-way ANOVA test followed by Turkey’s comparison were used to detect differences between groups. Correlations between IgG OD values and titres in the ELISAs and faecal egg counts [expressed in eggs per gram (EPG)] from the KK test results were determined by Spearman’s correlation coefficient after natural log transformation ln(Y + 1), where ‘Y’ represents EPG. Comparisons of data with a *p* value <0.05 were considered statistically significant.

## 3 Results

### 3.1 Demographic and clinical characteristics

A total of 450 participants were included in the current study, with 100 each from Bugoto, Bwondha, Musubi, and Bukizibu and 50 from Igeyero (Table 1). These subjects included 198 males and 252 females. The median age of the participants was 14 years, ranging from 5 to 78 years, with the 10–19 years age group predominating in the study population (*n* = 266, 59.1%). Approximately one-third of the individuals were infected with *S. mansoni* (36.4%) or hookworms (36.9%), as confirmed by the KK method. Among the participants with schistosome infection, the median EPG of the faecal egg burden was 31.7 (range 4–3,246), with 67.5, 20.5 and 10.8% of the participants having light (<100 EPG), moderate (100–399 EPG) and heavy (≥400 EPG) infections, respectively (Supplementary Table S1). Among the hookworm-infected individuals, the majority (*n* = 166, 98.8%) had a light infection (EPG: 1–1,999) (Supplementary Table S1). Seventy-two subjects were co-infected with *S. mansoni* and hookworms. In addition, 0.2, 1.3, and 7.3% of the subjects were infected with *Ascaris*, *Trichuris* and other helminths, respectively (Table 1).

**Table 1:** Sociodemographic characteristics and helminth infection status of study participants in five schistosomiasis endemic villages in Mayuge District, Uganda.

Characteristics	Bugoto	Bwondha	Musubi	Bukizibu	Igeyero	Total
	*N* = 100	*N* = 100	*N* = 100	*N* = 100	*N* = 50	*N* = 450
Age groups (years)						
<10	20 (20.0)	6 (6.0)	6 (6.0)	20 (20.0)	0 (0.0)	52 (11.6)
10–19	46 (46.0)	55 (55.0)	68 (68.0)	47 (47.0)	50 (100.0)	266 (59.1)
20–29	1 (1.0)	7 (7.0)	8 (8.0)	10 (10.0)	0 (0.0)	26 (5.8)
30–39	10 (10.0)	18 (18.0)	11 (11.0)	11 (11.0)	0 (0.0)	50 (11.1)
40–49	12 (12.0)	10 (10.0)	4 (4.0)	6 (6.0)	0 (0.0)	32 (7.1)
50–59	9 (9.0)	2 (2.0)	2 (2.0)	5 (5.0)	0 (0.0)	18 (4.0)
>60	2 (2.0)	2 (2.0)	1 (1.0)	1 (1.0)	0 (0.0)	6 (1.3)
Sex						
Male	43 (43.0)	42 (42.0)	47 (47.0)	42 (42.0)	24 (48.0)	198 (44.0)
Female	57 (57.0)	58 (58.0)	53 (53.0)	58 (58.0)	26 (52.0)	252 (56.0)
*Schistosoma mansoni*						
Yes	49 (49.0)	49 (49.0)	40 (40.0)	23 (23.0)	3 (6.0)	164 (36.4)
No	51 (51.0)	51 (51.0)	60 (60.0)	77 (77.0)	47 (94.0)	286 (63.6)
Hookworm						
Yes	28 (28.0)	38 (38.0)	38 (38.0)	51 (51.0)	13 (26.0)	168 (37.3)
No	72 (72.0)	62 (62.0)	62 (62.0)	49 (49.0)	37 (74.0)	282 (62.7)
*Ascaris*						
Yes	0 (0.0)	0 (0.0)	0 (0.0)	1 (1.0)	0 (0.0)	1 (0.2)
No	100 (100.0)	100 (100.0)	100 (100.0)	99 (99.0)	50 (100.0)	449 (99.8)
*Trichuris*						
Yes	0 (0.0)	6 (6.0)	0 (0.0)	0 (0.0)	0 (0.0)	6 (1.3)
No	100 (100.0)	94 (94.0)	100 (100.0)	100 (100.0)	50 (100.0)	444 (98.7)
*Others (Taenia*, *Heterophydes, E. vermicularis*, *Hymenolepis nana)*						
Yes	6 (6.0)	6 (6.0)	6 (6.0)	10 (10.0)	5 (10.0)	33 (7.3)
No	94 (94.0)	94 (94.0)	94 (94.0)	90 (90.0)	45 (90.0)	417 (92.7)

### 3.2 COVID-19 vaccination status

**Figure 2:**
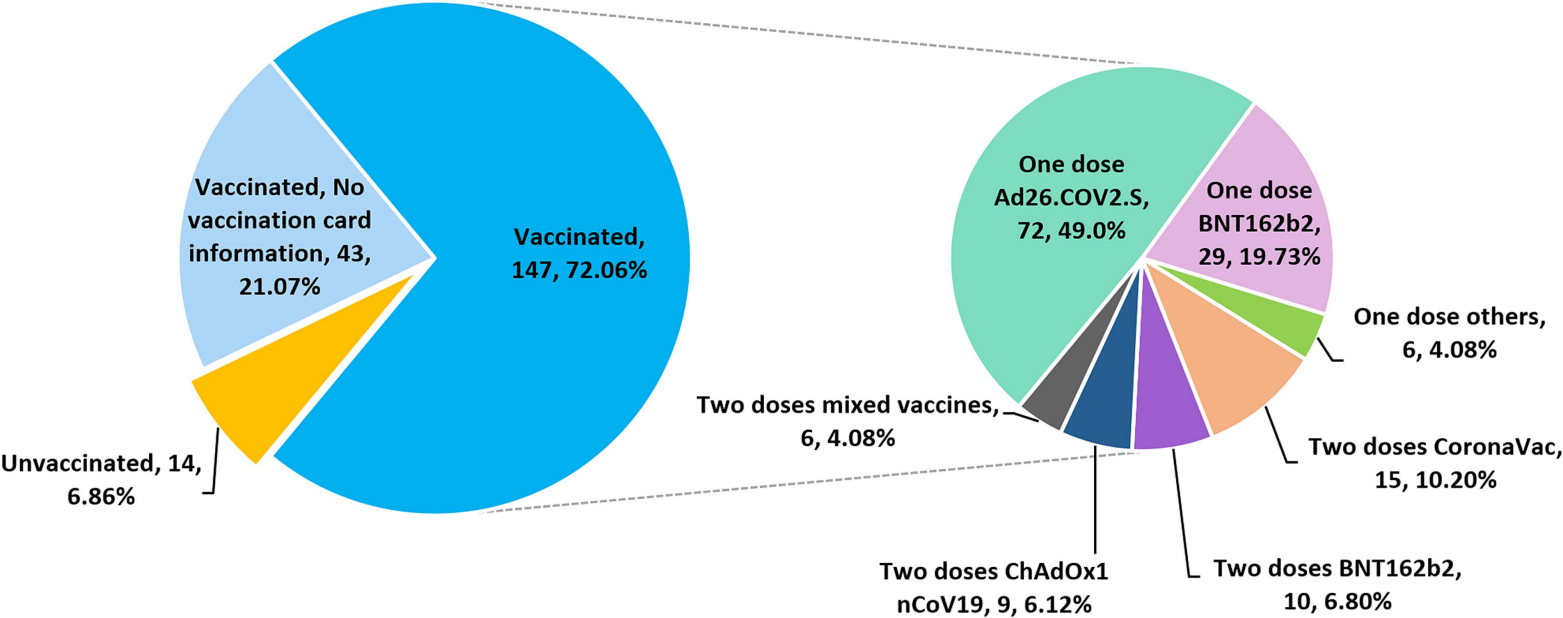
Pie charts showing the COVID-19 vaccination status and regimens of 204 subjects who participated in the vaccination survey.

A total of 204 participants were asked about their COVID-19 vaccination status. Among these subjects, 190 (93.14, 95% CI, 88.8–95.9%) self-reported having received at least one dose of COVID-19 vaccine (Figure 2). Among the 147 participants who provided detailed vaccination information, most were vaccinated with a single-dose of Ad26.COV2.S (*n* = 72, 49.0%), followed by a single dose of BNT162b2 (*n* = 29, 19.73%), whereas 6.12, 6.8, 10.2 and 4.08% of the subjects received two doses of ChAdOx1 nCoV-19, BNT162b2, CoronaVac and mixed COVID-19 vaccines, respectively (Figure 2). A mixed-dose regimen occurred when the first and second doses were of different vaccine brands. No booster dose was given to the vaccinees who completed a primary 2-dose BNT162b2 mRNA vaccine series. Based on the available vaccination information collected, the date of vaccine administration ranges from September 10, 2021 to September 28, 2022.

### 3.3 Anti-SARS-CoV-2 spike protein S1 IgG responses

**Figure 3:**
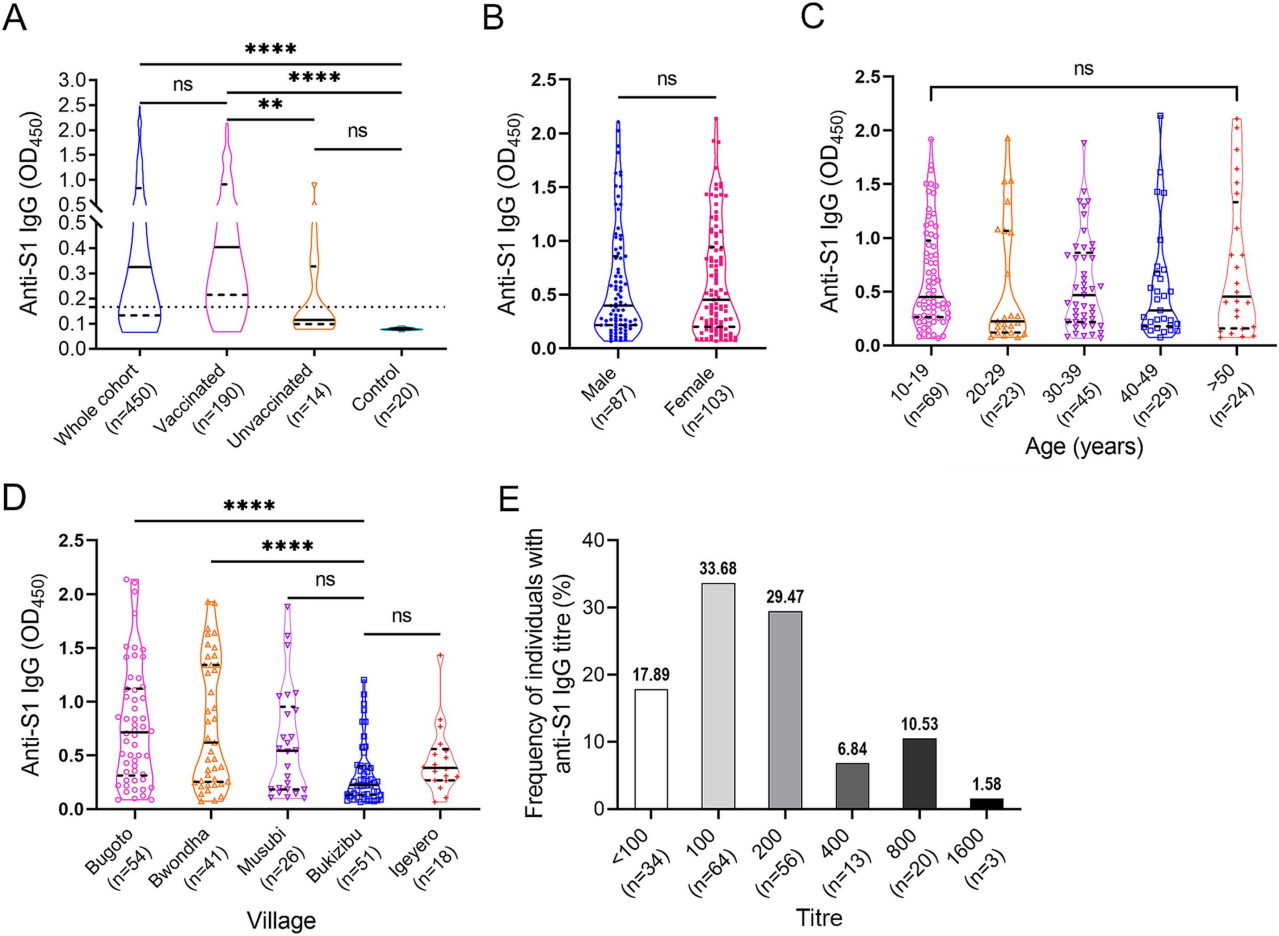
SARS-CoV-2 specific anti-S1 IgG responses determined by ELISA. (A) IgG levels in the whole cohort (*n* = 450), vaccinated (*n* = 190), unvaccinated (*n* = 14) and control (*n* = 20) groups. The Kruskal-Wallis test followed by Dunn’s multiple comparison test was used to determine significant differences between groups (ns = no significant difference, **p < 0.001, ****p < 0.0001). (B) Comparison of anti-S1 IgG levels between male and female vaccinees. *p* value was determined using the Mann–Whitney *U* test (ns = no significant difference). (C) Comparison of anti-S1 IgG levels across the five age groups. (D) Comparison of anti-S1 IgG levels among the five villages. *p* values were determined by one-way ANOVA followed by Turkey’s comparison (****p < 0.0001). (E) Frequencies of COVID-19 vaccinees (*n* = 190) with different anti-S1 IgG titre levels. Panels A–D, truncated violin plots are shown. The horizontal black lines indicate median values, and the horizontal black dotted lines indicate the interquartile range (IQR).

Specific anti-S1 IgG levels were determined in all the serum samples via ELISA at a dilution of 1:100 (Figure 3A). Within the cohort, 314 (69.78%) subjects had a positive anti-S1 IgG response. Anti-S1 IgG levels were significantly higher in the vaccinated group than those in the unvaccinated and control groups (*p* < 0.01 and < 0.0001, respectively). No difference in anti-S1 IgG levels was observed between the unvaccinated and control groups. However, 4 out of 14 non-vaccinated subjects showed a weak positive reaction. There was no difference in anti-S1 IgG levels between male and female vaccinees (*p* > 0.05) (Figure 3B) or between different age groups (*p* > 0.05 in all group comparisons) (Figure 3C). Among the five villages, vaccinees in Bugoto and Bwondha had significantly higher IgG levels than those in Bukizibu (*p* < 0.0001) (Figure 3D). In the vaccinated group, the anti-S1 IgG titre ranged from <100 to 1,600. Most COVID-19 vaccinees had an anti-S1 IgG titre of 100 (33.68%), followed by 200 (29.47%), while 17.89% of vaccinated subjects had an anti-S1 IgG titre below 100 (Figure 3E). Only 6.84, 10.53 and 1.58% of the subjects had anti-S1 IgG titres of 400, 800 and 1,600, respectively.

### 3.4 Anti-S1 IgG responses in adolescents receiving a single dose of BNT162b2

**Figure 4:**
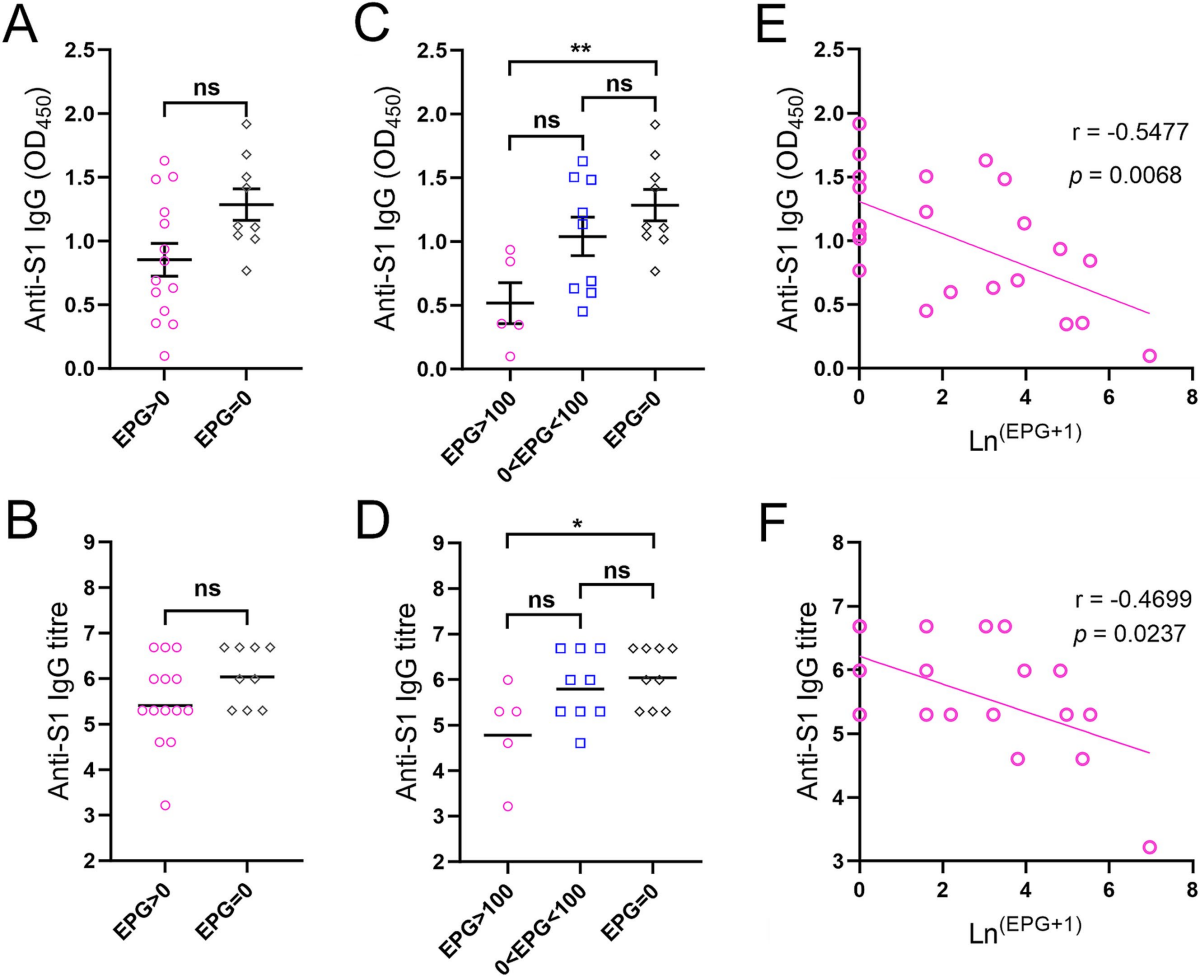
Anti-S1 IgG responses in adolescents receiving a single dose of the BNT162b2 vaccine two and a half months before the helminth survey. (A) Comparison of IgG levels between KK-positive (*n* = 14) and KK-negative subjects (*n* = 9) (serum dilution: 1:100). (B) Comparison of IgG titres between KK-positive (*n* = 14) and KK-negative subjects (*n* = 9). *p* values were determined using the Mann–Whitney *U* test (ns = no significant difference). (C,D) Comparison of anti-S1 IgG levels (C) and titres (D) between participants with moderate or heavy *S. mansoni* infection (*n* = 5), light *S. mansoni* or hookworm infection (*n* = 9) and KK-negative subjects (*n* = 9) (serum dilution: 1:100). IgG titres were logarithmically transformed. *p* values were determined by one-way ANOVA followed by Turkey’s comparison test (ns = no significant difference, *p < 0.05, **p < 0.01). (E,F) Correlations between anti-S1 IgG levels (E) and titres (F) from ELISAs and faecal egg count determined by the KK method in subjects receiving a single dose of the BNT162b2 vaccine (*n* = 23) were calculated using Spearman’s correlation coefficient.

Twenty-three adolescents (aged 12–17 years) who received a single dose of BNT162b2 were stratified by faecal egg count (Supplementary Table S2). Anti-S1 IgG levels and titres measured two and a half months after vaccination showed no difference between KK-positive (EPG > 0, *n* = 14) and KK-negative (EPG = 0, *n* = 9) subjects (Figures 4A,B). However, IgG levels/titres were significantly lower in subjects with moderate or heavy *S. mansoni* infection (*n* = 5) than in KK-negative subjects (*p* < 0.01 and < 0.05, respectively) (Figures 4C,D), whereas no difference was observed between subjects with light infection (1–99 EPG for *S. mansoni*, 1–999 EPG for hookworms, *n* = 9, i.e., five individuals with *S. mansoni* infection, three individuals with hookworm infection, and one individual with *S. mansoni* and hookworm co-infection) and KK-negative individuals (*n* = 9). Within the sub-cohort, anti-S1 IgG antibody levels (OD values) and log-transformed IgG titres were inversely correlated with faecal egg counts determined by the KK method (*r* = −0.5477, *p* = 0.0068 and *r* = −0.4699, *p* = 0.0237, respectively) (Figures 4E,F).

### 3.5 Anti-S1 IgG isotypes in the two vaccinated subgroups

**Figure 5:**
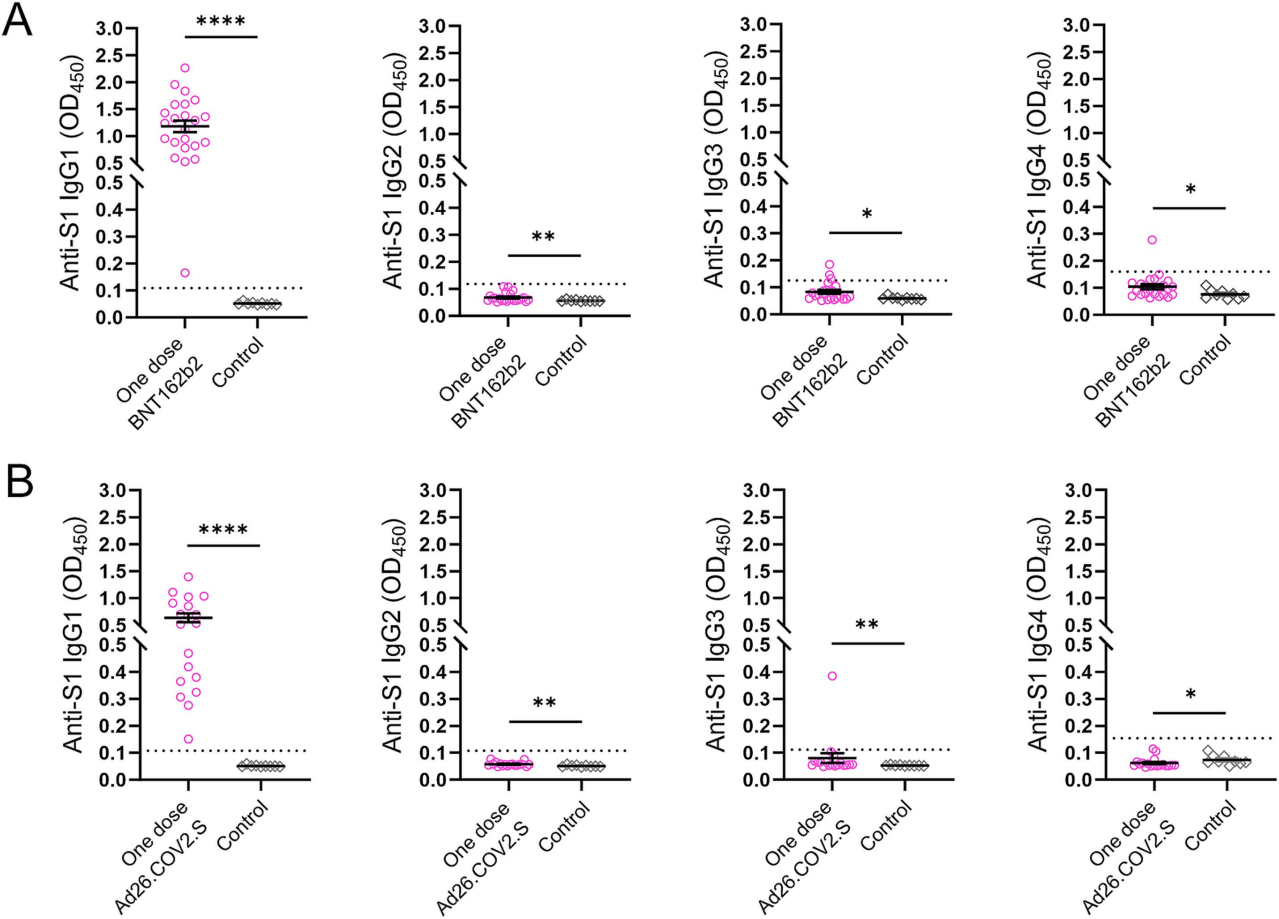
Anti-S1 IgG antibody subclasses in the two subsets after COVID-19 vaccination. (A) Subjects (*n* = 23) who received a single dose of the BNT162b2 vaccine. (B) Subjects (*n* = 18) who received a single dose of the Ad26.COV2.S vaccine. Serum samples (*n* = 9) collected prior to the COVID-19 outbreak served as controls. The dotted lines indicate cut-off OD values. *p* values were determined using the Mann–Whitney *U* test (*p < 0.05, **p < 0.01, ****p < 0.0001).

Four IgG subclasses targeting the SARS-CoV-2 S1 protein were measured in 41 participants including subjects (*n* = 23) who received a single dose of the BNT162b2 mRNA vaccine two and a half months prior to recruitment and individuals (*n* = 18) who were vaccinated with a single dose of Ad26.COV2.S approximately 4 months prior to sampling (Figure 5). All participants in both vaccination groups were positive for anti-S1 IgG1 antibody, whereas three subjects and one individual in the groups receiving a single dose of BNT162b2 and Ad26.COV2.S, respectively, were IgG3 positive (Figures 5A,B).

## 4 Discussion

SARS-CoV-2, a novel virus that emerged in late 2019 and caused COVID-19, precipitated an unprecedented global health and economic crisis. The pandemic led to the rapid development and deployment of novel and effective COVID-19 vaccines. Following the global introduction of COVID-19 vaccination, it was important to assess the immunomodulatory effect of helminth infections on COVID-19 vaccine-induced immune responses and vaccine efficacy in LMICs (33, 34). In the Mayuge District, Eastern Uganda, *S. mansoni* and hookworms are the most common helminth species. The average schistosomiasis prevalence in the five study villages was 36.4%, which is higher than the national prevalence recorded in 2018 (26.5%) (25). In the current study, the three lakeshore villages, Bugoto, Bwondha and Musubi recorded similar schistosomiasis prevalence rates (~50%), which are higher than those in Bukizibu (23%) and Igeyero (6%). This finding is consistent with the observation that *S. mansoni* prevalence rates are higher in communities located 5 km or less from the shores of Lake Victoria or Lake Albert than in those located more than 5 km away (35). The prevalence data from the three lakeshore villages were similar to those of a previous study conducted before the COVID-19 outbreak (36), suggesting that the pandemic did not significantly affect schistosomiasis transmission in these areas. However, the persistence of schistosomiasis in these villages despite prolonged mass drug administration (MDA) and education was probably due to low MDA coverage and compliance as well as common misconceptions about schistosomiasis (37, 38).

Few studies have assessed COVID-19 coverage and serum antibodies against the SARS-CoV-2 spike protein in rural communities with co-endemic helminth infections. Here, we investigated COVID-19 vaccination status, the uptake of different COVID-19 vaccine brands, and IgG antibody responses to the SARS-CoV-2 spike S1 protein in 204 villagers who participated in a cross-sectional helminth survey in Mayuge District. One hundred ninety of the 204 individuals who participated in the vaccination survey received at least one dose of the COVID-19 vaccine, indicating a high vaccination coverage in the cohort. The highest uptake was recorded for the single-dose Ad26.COV2.S vaccine (72/147, 49.0%) followed by the BNT162b2 vaccine (one dose, 19.7%; two doses, 6.8%). Additionally, the percentages for the uptake of two-dose of ChAdOx1 nCoV-19 and CoronaVac vaccines were 6.12% and 10.20%, respectively. The variability in the uptake of different COVID-19 vaccine brands may be due to personal preferences or vaccine availability at immunization clinics. Although there was a high acceptance rate, 81% of COVID-19 vaccinees had anti-S1 IgG titres ≤200, indicating that the antibody levels had declined in most vaccinees by the time of sampling. The majority of participants had not completed the two-dose primary BNT162b2 mRNA vaccination series. The low antibody levels and rapid decline probably underscore the overall impact of helminth infections on the performance of all vaccines and the importance of receiving the second dose of two-dose COVID-19 vaccines. At the time of the study, although the national level case data showed that COVID-19 prevalence in Uganda was low (0.3%), it was reported that the SARS-CoV-2 infection rate in unvaccinated individuals was high in rural Eastern Uganda (39). In this study, four of the 14 unvaccinated participants were anti-S1 IgG positive, which may be due to a previous SARS-CoV-2 infection or cross-reactivity to common cold coronaviruses circulating in the region (8, 40).

Among the participants who received a single dose of the BNT162b2 vaccine within a narrow time window (Supplementary Table S2), an inverse correlation was observed between the faecal egg count and anti-S1 antibody titre. Compared to KK-negative individuals, those with moderate or heavy *S. mansoni* burden had significantly lower anti-S1 IgG levels/titres. These results indicate an impaired humoral immune response to the BNT162b2 vaccine in the group with an EPG > 100, and more broadly, imply an infection intensity-dependent immunomodulatory effect on the immune responses elicited by COVID-19 vaccines. Our results are thus at least partially consistent with those of previous studies, e.g., Musaigwa *et al* reported that chronic *S. mansoni i*nfection can reduce the persistence of antibody responses in humans and mice immunized with poliovirus vaccines (41). Similarly, schistosome infection also negatively affects antibody levels/titres after hepatitis B (HepB) vaccination (19, 42). In contrast, infection with *Heligmosomoides polygyrus bakeri*, a gastrointestinal-restricted murine hookworm, has been recently shown to have limited effects on B-cell responses induced by an mRNA vaccine against SARS-CoV-2 (43). Nevertheless, the potential mechanisms responsible for the feeble antibody responses to the S1 protein in vaccinated participants with moderate-to-heavy schistosome infection are not known. Possible underlying mechanisms include: (i) chronic schistosome infection, which typically induces a polarized Th2 immune response, creating a cytokine environment that potentially suppresses T follicular helper (Tfh) responses, which are critical for providing ‘B-cell help’ in response to vaccination (19). (ii) *S*. *mansoni* infection can potentially induce the death of antibody-producing plasma cells in the bone marrow (41). Further mechanistic studies are needed to address this issue. Nevertheless, there is also accumulating evidence that deworming could enhance/restore the immune response to vaccines against a variety of pathogens (41, 44, 45). To ensure adequate immune protection against SARS-CoV-2 infection, individuals infected with schistosomes should complete their primary COVID-19 vaccine course at least, and ideally, receive a booster dose. A ‘treat and vaccinate’ strategy of treatment with praziquantel (PQZ) could also be implemented in these individuals prior to vaccination.

This study has several limitations. First, the small sample size and cross-sectional design of the study did not allow us to perform statistical modeling to adjust for potential confounders (e.g., age, sex, vaccine type, and helminth species) or to perform multiple correction testing. Second, blood sampling at only one time point likely missed important changes in serum antibody concentrations during the post-vaccination period. In addition, sample collection during the survey occurred at different times but not at a standard time point post-vaccination. Third, no previous exposure to coronaviruses was investigated in this study. We cannot completely rule out the possibility that some of the anti-S1 IgG responses detected in the cohort studied were due to a previous SARS-CoV-2 or common hCoV infection (5, 8). Finally, we cannot conclude whether the observed antibody responses induced by single-dose COVID-19 vaccination provide protection because we did not assess the functional activity of the antibodies in a virus neutralization assay.

## 5 Conclusion

The outbreak of COVID-19 did not significantly alter the prevalence of schistosomiasis in three lakeshore villages in Mayuge District. Our study confirmed that the acceptance rate of COVID-19 vaccination in these schistosomiasis-endemic areas was high at the time of investigation but the anti-S1 IgG levels and titres in these vaccinees were low. Moderate-to-heavy schistosome infection suppressed COVID-19 vaccine-induced antibody responses in a subgroup of participants who received a single dose of the BNT162b2 mRNA vaccine. However, these results need to be validated in other studies with larger sample sizes in different endemic settings. Our findings therefore call for further research on the immunomodulatory effect of helminth infections on the efficacy of COVID-19 vaccination, which may inform the implementation of a ‘treat and vaccinate’ policy in LMICs with a heavy helminth burden.

## Data Availability

All data supporting the findings of this study are included in the article/Supplementary material. Further inquiries can be directed to the corresponding author.
